# Autophagy protein NRBF2 has reduced expression in Alzheimer’s brains and modulates memory and amyloid-beta homeostasis in mice

**DOI:** 10.1186/s13024-019-0342-4

**Published:** 2019-11-27

**Authors:** Véronik Lachance, Qian Wang, Eric Sweet, Insup Choi, Cui-Zan Cai, Xu-Xu Zhuang, Yuanxi Zhang, Jessica Li Jiang, Robert D. Blitzer, Ozlem Bozdagi-Gunal, Bin Zhang, Jia-Hong Lu, Zhenyu Yue

**Affiliations:** 1 Department of Neurology, The Friedman Brain Institute, Icahn School of Medicine at Mount Sinai, New York, NY 10029 USA; 2 Department of Genetics and Genomic Sciences, Icahn School of Medicine at Mount Sinai, New York, NY 10029 USA; 3 Departments of Psychiatry and Pharmacological Sciences, Icahn School of Medicine at Mount Sinai, New York, NY 10029 USA; 4 Present Address: Department of Biology, West Chester University, West Chester, PA 19383 USA; 5 State Key Laboratory of Quality Research in Chinese Medicine, Institute of Chinese Medical Sciences, University of Macau, Taipa, Macau SAR China; 6 Department of Neuroscience, Icahn School of Medicine at Mount Sinai, New York, NY 10029 USA; 7 Present Address: Department of Psychiatry, Rutgers New Jersey Medical School, Newark, NJ 07103 USA

## Abstract

**Background:**

Dysfunctional autophagy is implicated in Alzheimer’s Disease (AD) pathogenesis. The alterations in the expression of many autophagy related genes (ATGs) have been reported in AD brains; however, the disparity of the changes confounds the role of autophagy in AD.

**Methods:**

To further understand the autophagy alteration in AD brains, we analyzed transcriptomic (RNAseq) datasets of several brain regions (BA10, BA22, BA36 and BA44 in 223 patients compared to 59 healthy controls) and measured the expression of 130 ATGs. We used autophagy-deficient mouse models to assess the impact of the identified ATGs depletion on memory, autophagic activity and amyloid-β (Aβ) production.

**Results:**

We observed significant downregulation of multiple components of two autophagy kinase complexes *BECN1-PIK3C3* and *ULK1/2-FIP200* specifically in the parahippocampal gyrus (BA36). Most importantly, we demonstrated that deletion of *NRBF2*, a component of the BECN1-PIK3C3 complex, which also associates with ULK1/2-FIP200 complex, impairs memory in mice, alters long-term potentiation (LTP), reduces autophagy in mouse hippocampus, and promotes Aβ accumulation. Furthermore, AAV-mediated *NRBF2* overexpression in the hippocampus not only rescues the impaired autophagy and memory deficits in NRBF2-depleted mice, but also reduces β-amyloid levels and improves memory in an AD mouse model.

**Conclusions:**

Our data not only implicates *NRBF2* deficiency as a risk factor for cognitive impairment associated with AD, but also support the idea of *NRBF2* as a potential therapeutic target for AD.

## Background

Alzheimer’s disease (AD) is the leading cause of dementia affecting our elders and the seventh cause of death worldwide. While genetic variants contribute to a subset of AD cases, aging persist to be the primary risk factor for AD. In addition, the pathological hallmarks of AD are the excessive β-amyloid deposits (Aβ) and intraneuronal neurofibrillary tangles containing hyperphosphorylated-tau (pTau) [1–3]. The aberrant accumulation of Aβ and pTau suggests a failure of protein handling system during the course of the disease. In fact, loss of the proteostasis network — including the autophagy pathway — is implicated in the pathogenesis of AD [4–8].

Over the past decades, many studies have documented the dysregulation of autophagy in AD postmortem brains and experimental models. Early ultrastructural analysis of AD brains showed accumulation of autophagic vacuoles (AVs) in dystrophic neurites [9] and examination of autophagy pathway showed upregulation of mTOR activity, a negative regulator of autophagy signaling [10], and reduced expression of Beclin 1, a core component of class III PI3-kinase (PIK3C3) that controls autophagy initiation [11], therefore suggesting that autophagy is impaired in AD. However, a genome-wide analysis indicated a transcriptional upregulation of autophagy in entorhinal cortex of AD patients [12], and others reported hyperactivation of AMPK, a positive autophagy signaling kinase, thus supporting an enhanced autophagic activity in AD [13–15]. A recent finding showed that hippocampal neurons isolated from AD subjects contained greater expression of genes or proteins related to autophagosomes and lysosomes biogenesis. However, the same study suggests an impediment of autophagy flux despite the enhanced autophagy biogenesis [16]. Thus, the available evidence for autophagy alteration in AD appears conflicting, obscuring the role of autophagy in the disease’s onset and progression. It is conceivable that multiple factors may contribute to the discrepancies in these results, such as the small sample size, the disease stages, the distinct brain regions and the ATGs examined. Hence, studies with increased sample size and improved approaches are necessary to comprehend the precise function of autophagy in AD. Herein, we examined the expression of over 100 autophagy related (ATG) genes in multiple brain regions from more than 200 AD postmortem brains. Our analysis revealed significant downregulation of genes encoding autophagy kinase complexes in the parahippocampal gyrus and hippocampus. Our data suggest that loss of *NRBF2* functions in the hippocampus impairs memory in mice and may contribute to the cognitive impairment associated with AD. Our study also supports NRBF2 as a potential therapeutic target.

## Methods

### Bioinformatics analysis

A list of 130 core ATGs was manually curated based on literature reviews [17, 18] and public database (www.tanpaku.org/autophagy/) [19]. Expression of these genes was examined at the mRNA level in multiple brain regions of healthy control and AD patient samples from the Mount Sinai Brain Bank https://www.synapse.org/#!Synapse:syn3157743) [20]. Differential gene analysis was performed using Bioconductor R Limma Package [21] with Benjamini-Hochberg correction for multiple testing. Spearman correlation analysis was performed to examine the relationship between ATG expression and CDR score. Adjusted *p* value< 0.05 was considered statistically significant.

### Animals

Subjects were housed in groups of two to five. Food and water were supplied ad libitum in an animal facility with a regular 12 h light/dark cycle (light on at 7:00 A.M.). Tails were cut and ears were notched when pups were 7–14 days for genotyping and identification purposes respectively. NRBF2-KO mouse genotyping was performed as mentioned in [22] and the standard protocol of Jackson Laboratory was used for the 5XFAD mouse genotyping (stock number 008730). Mice were weaned at 21 days. Unless mentioned, mice used in this study were from three different cohorts, aged around 3–4 months. For all test conditions, the male:female ratio was ~ 1:1 and compare to WT littermate controls.

### Reagents

NuPAGE® MOPS SDS Running Buffer (20X), (#NP0001–02), NuPAGE® MES SDS Running Buffer (20X), (#NP0002–02), NuPAGE™ 4–12% Bis-Tris Protein Gels, 1.0 mmX15well, (#NP0323BOX), NuPAGE™ 4–12% Bis-Tris Protein Gels, 1.0 mmX26well, (#WG1403BOX) and PierceTM BCA Protein Assay Kit (#23225) and ProLong Diamond antifade mountant with or without DAPI (#P36962 or #P36961) were from Thermo Scientific. Western Lightning Plus ECL (#NEL105001EA) was from PerkinElmer. Immobilon®-FL Transfer membrane 0.45 um, Polyvinylidene Difluoride (PVDF) membrane (#ISEQ00010) was from Merck Millipore. HyBlot CL films (#E3012) were from Denville Scientific, Inc. Non-fat dry milk (#M0841) was from Lab Scientific. Protease and Phosphatase inhibitors tablets (#88669) were from Thermo Scientific. Dynabeads Protein G was from Novex (Life Technologies, #10004D). Ponceau S Solution (#P7170) was from Sigma-Aldrich. OCT compound (#23–730-571) and microscope slides (# 12–550-15) were from Fisher Scientific. Liquid blocker pap pen (#71310) was from Electron Microscopy Sciences (EMS).

### Antibodies

#### Immunoblotting

AMPKα (D5A2) Rabbit mAb (#5831, 1:1000), phospho- AMPKα (Thr172) (40H9) Rabbit mAb (#2535, 1:500), Raptor (24C12) Rabbit mAb (#2280, 1:500), phospho-Raptor (Ser792) Rabbit polyclonal antibody (pAb) (#2083, 1:500), mTOR (7C10) Rabbit mAb (#2983, 1:500), phospho-mTOR (Ser2481) Rabbit pAb (#2974, 1:500), 4E-BP1 (#9452, 1:500), phospho-4E-BP1 (Thr37/46) (236B4) Rabbit mAb (#2855, 1:1000), ULK1 (D8H5) Rabbit mAb (#8054, 1:500), FIP200 (D10D11) Rabbit mAb (#12436, 1:250), LC3B Rabbit pAb (#2775, 1:1000) and Rabbit (DA1E) mAb IgG XP® Isotype Control (#3900) were from Cell Signaling Technology. Goat anti-Rabbit IgG-HRP pAb (sc-2004, 1:1000) were from Santa Cruz Biotechnology, Inc. NRBF2 Rabbit pAb (A301-851A) was from Bethyl Laboratories, Inc. P62 Guinea pig pAb (#GP62-C, 1:4000) was from PROGEN. P62 Guinea pig pAb (PM066, 1:4000) was from Medical and Biological Laboratories Co., LTD. (MBL). Goat anti-Mouse IgG-HRP (#A28177, 1:1000), goat anti-Guinea Pig IgG-HRP (#A18775, 1:1000) antibodies were from Thermo Scientific. PSD95 (6G6-1C9) mAb (#MA1–045, 1:1000) was from Thermo Scientific. VAMP2 Rabbit pAb (#104202, 1:5000) was from Synaptic Systems.

#### Immunohistochemistry

Phophos- AMPKα (Thr172) Rabbit pAb (#AP0432, 1:200 IHC) was from ABclonal Technology. β-Amyloid (D54D2) Rabbit monoclonal antibody (mAb) (#8243, 1:500) and cleaved Caspase-3 (Asp175) Rabbit pAb (#9661, 1:100) were from Cell Signaling Technology. Goat anti-Rabbit IgG-Alx647 (#A21246, 1:500) antibody was from Thermo Scientific.

### Perfusion and Cryo-sectioning

Mice were transcardially perfused with 25–30 ml (6–7 ml/min.) of ice-cold PBS 1X to remove excess blood, then perfused with 25–30 ml of ice-cold 4% paraformaldehyde. After perfusion, the brain was removed from the skull and fixed overnight at 4 °C in 15 ml falcon tube filled with 4% PFA. The next morning, the brain were washed 3x with 15 ml of ice-cold PBS 1X and incubate with 15 ml of 30% sucrose solution for at least 24 h or up until the brain has sunk at the bottom of the tube. Left and right sagittal hemisphere were divided, embedded in OCT compound, gradually froze in liquid nitrogen, and stored at − 80 °C. Cryo-sectioning was performed using Leica CM3050 S cryostat. 40 μm sagittal sections were conserved at − 20 °C in anti-freezing medium (25% glycerol, 30% ethylene glycol, 50 mM phosphate buffer pH 7.4).

### Heat-induced epitope retrieval

The protocol used is based on the following reference [23]. Briefly, sections were wash 3 × 10 min. With 500 μl PBS 1X at RT on microscope slides and allowed to dry for 20–30 min. in the dark. Next, we incubate the slides in pre-warmed citrate buffer (10 mM Sodium Citrate dihydrate pH 6.0, 0.05% Tween 20) in a water bath heated at 65 °C for 45 min. After incubation, slides were washed 3 × 10 min. at RT with PBS 1X while shaking. A final wash was executed for 10 min. at room temperature (RT) with PBS 1X containing 0.1% Triton-X-100.

### Immunofluorescence and confocal imaging

Brain sections were encircled with liquid blocker pap pen and blocked with 150 μl/section PBS containing 5% goat serum and 0.1% Triton X-100 for 1 h at RT. Sections were incubated in a humid chamber with 100 μl of primary antibody diluted in blocking buffer overnight at 4 °C. After washing 3 × 10 min. With 150 μl PBS, sections were incubated with 100 μl Alexa-conjugated secondary antibody for 1 h at RT. After 4 washes with PBS, sections were mounted with ProLong Diamond antifade reagent. Sections were examined under Carl Zeiss upright confocal microscope (LSM780 system). Images were taken sequentially with 40X oil immersion objective lens at RT. Single or tile scan acquisitions were performed by Zen2012 software.

### Confocal image analysis

Basic Intensity Quantification was performed with Image J. RGB pictures were converted into single 16-bit grayscale images. A duplicate of the grayscale picture was generated and further processed into a binary picture. The background was subtracted with the rolling ball (radius of 50.0 pixels) tool with light background and sliding paraboloid options selected. The threshold was finally adjusted to highlight all the structures having signal. Next, we calibrated the scale, set the measurements, and redirect it to the original grayscale image. The particles were analyzed and stated as mean gray intensity over the total area (μm^2^). We finally normalized these data to the WT mean values and reported the fold change.

### Tissue homogenization

Once the tissue harvested and flash frozen, 400 μl of homogenization buffer (0.32 M sucrose, 1 mM NaHCO_3_, 20 mM HEPES, 0.25 mM CaCl_2_, 1 mM MgCl_2_ and protease/phosphatase inhibitors) were added to 0.1 g of tissue and homogenized with blue pestle and cordless pestle motor in 1.5 or 2.0 ml Eppendorf tube. Using insulin syringe, 20 up and down were performed to disrupt the tissue. The homogenates were incubated for 30 min. at 4 °C using end over end mixing. Then the samples were centrifuged at 1500×g for 10 min. at 4 °C, the supernatant harvested, and the pellet discarded. Samples were diluted 1:10 and protein concentration was measured.

### Immunoprecipitation

Immunoprecipitations (IPs) were performed using 150 μg of proteins extracted from hippocampal homogenates diluted in 300 μl of homogenization buffer. Anti-ULK1 antibody (1:150, v/v) or isotype control (same concentration than ULK1 Ab) were added to each tube and incubated overnight at 4 °C with end over end agitation. The next morning, 30 μL of Dynabeads Protein G was added, followed by an 1 h. incubation at 4 °C. Samples were then centrifuge 1 min at 4000 RPM in a microcentrifuge and washed three times with 1 mL of homogenization buffer, immunoprecipitated proteins were eluted by addition of 30 μL of 4X SDS sample buffer, followed by a 10 min. Incubation at 95 °C. Initial lysates and immunoprecipitated proteins were analyzed by SDS-PAGE and immunoblotting with specific antibodies.

### Immunoblotting

All biological samples have been analyzed at least in duplicate in two independent experiments. Samples were diluted to 1 mg/ml with buffer and SB4X denaturation buffer (200 mM 4X Tris-HCl/SDS ph 6.8, 8% SDS, 400 mM DTT, 40% glycerol (v/v) 0.4% Bromophenol Blue) diluted to 1X. Samples were denatured at 95 °C for 10 min and spin at RT for 30s at 14000 rpm. 10μg of protein samples were separated on 4–12% Bis-Tris NuPAGE gels for 70-80 min. at 150 V at room temperature (RT) using 1X MOPS or 1X MES buffer (Invitrogen). The proteins were transferred to a PVDF membrane for 1 h at 100 V at 4 °C. The membranes were dried and stained for 10 min. With Ponceau S. Excess stain was removed for 2 min. With Milli-Q water. The membranes were scanned, cut and block in TBS 1X containing 0.1% Tween 20 (TBST) and 5% non-fat dry milk for 1 h at RT. Primary antibodies were applied, and membranes were incubated overnight at 4 °C. The membranes were washed 3 × 8 min. With TBST. Secondary antibodies were applied, and membranes were incubated for 1 h at RT. The membranes were washed 3 × 8 min. With TBST and twice with TBS 1X. The proteins were visualized using ECL detection kit.

### Immunoblot membrane stripping

After phospho-antibodies detection, membranes were washed once during 10 min. in distilled water to remove ECL. Membranes were incubated 3 × 10 min. in NaOH (0.2 M) solution to strip off the antibodies. Membranes were finally incubated in TBST buffer for 10 min. and the blotting procedure was started over.

### Densitometry analysis

Western blot quantification was performed based on the recommendations of Gassmann et al. [24]. All quantified immunoblots were revealed using the same type of films and carefully exposed to avoid saturation. Films were scanned using an Epson Perfection v500 or v800 Photo scanner. Acquisition was performed at 600 dpi in 16-bits grayscale with auto-exposure and colour-correction options turned off. Images were analyzed using the ImageJ software. Lanes were selected and plotted using the ‘Gel analyzer’ functions. Peaks on the plots were individually closed to the background level of each lane using the Straight line’ tool and the enclosed area was measured using the ‘Wand’ tool.

### Stereotaxic surgery

Stereotaxic delivery of recombinant adeno-associated virus, serotype 9 (AAV) for expression of a mCherry or NRBF2-mCherry fusion protein under control of the CMV promoter was done as follows: mice were anesthetized with 2% isoflurane and 1 μl of virus for each hemisphere (∼5.8 × 10^10^ viral genomic copies) was injected at a rate of 0.2 μl/min using a Hamilton syringe, a micro pump, and stereotaxic instrument (Stoelting). Syringe remained in place for an additional 2 min. After completion of the injection. Coordinates for injection were as follows: − 2.0 mm anterior/posterior, ±1.5 mm medial/lateral, and − 1.75 mm dorsal/ventral. Viruses were produced and purified by Vigene Biosciences Inc. (Rockville, MD, USA).

### Mouse Aβ_42_ ELISA

Aβ_42_ level was quantify from hippocampal extracts. Fractions were analyzed in duplicate. Same protein amount was loaded into each well, and the plate was incubated overnight at 4 °C with gentle agitation. ELISA was performed according to the manufacturer’s instructions (#KMB3441, Thermo Scientific).

### Behavior studies

#### Object Location Task (OLT) [25]

On habituation day (day 1), mice were individually placed into an open-field box (40 × 40 cm) surrounded by 40 cm high walls made of transparent plastic and allowed to freely explore the arena for 5 min in an infrared-lit room. On training day (day 2), mice were placed into the previously explored box now containing two similar objects and allowed to explore for 10 min. On testing day (day 3), one object was moved forward, and the mice were placed back into the arena and allowed to explore for 10 min. Videos were recorded by the EthoVision video tracking system (Noldus, Wageningen, The Netherlands) and were manually scored. Object explorations were counted once the following criteria have been met: the snout of the mouse is oriented toward and close to the object and the animal’s body is beyond the object (no climbing). To assess object bias we evaluate the time spent sniffing each object on day 2 relative to the total time spent exploring (Time spent sniffing object 1/ Total time spent sniffing object 1 and 2 X 100%). Mice exhibiting an object bias score below 0.2 or above 0.8 were excluded. Discrimination ratio was calculated as follows: time spent sniffing the object divided by the total time spent sniffing both objects, Score equivalent to 0.5 indicates equal time spent exploring the displaced and familiar objects.

#### Contextual fear conditioning (CFC)

CFC experiments were conducted in sound attenuating chambers with automated stimulus delivery software (Med Associates, St. Albans, VT, USA). On training day, mice were exposed to a 218 s period of acclimation to the conditioning arena (context A) followed by three consecutive foot shocks (0.5 mA, 2 s, 100 s interval between shocks) and a final 30 s resting period. On testing day, mice were re-exposed to context A for 3 min. One hour after re-exposure to context A, mice were placed in a modified arena (context B) and allowed to explore for 3 min. Percentage time freezing was quantified by automated motion-sensitive software (Video Freeze; Med Associates).

#### Radial-arm maze (RAM) [26]

The maze consisted of eight arms (7.5 × 35 cm, 17.5 cm high walls) assembled radially around a circular starting platform. Mice were placed onto the starting platform and were free to enter the arms. Mice were tested until all eight arms were visited once. Each repeated entry in arm was counted as an error. Mice were trained on day 1 and tested on day 2.

#### Spontaneous Alternation Task (SAT) [25]

The Y-Maze (Maze Engineers) consisted of three gray acrylic closed arms measuring 35 cm L × 5 cm W × 20 cm H. Mice were placed in the center of the maze and were free to explore for 10 min. The number of alternation and the number of entries were recorded and scored by the EthoVision video tracking system (Noldus, Wageningen, The Netherlands). Percentage of alternation was calculated as the number of alternation/ (total number of entries - 2) × 100. Total number of entries was reported to control any potential hyperactivity.

### Hippocampal slice preparation and field electrophysiology

Hippocampal slices (350–400 μm) were prepared from NRBF2-deficient mice and wild type littermates. Slices were perfused with Ringer’s solution containing (in mM): NaCl, 125.0; KCl, 2.5; MgSO_4_, 1.3; NaH_2_PO_4_, 1.0; NaHCO_3_, 26.2; CaCl_2_, 2.5; glucose, 11.0, and bubbled with 95% O2/5% CO2, at 32 °C during extracellular recordings. Slices were maintained for 1–2 h prior to establishment of a baseline of field excitatory postsynaptic potentials (fEPSPs) recorded from stratum radiatum in area CA1, evoked by stimulation of the Schaffer collateral-commissural afferents (100 μs pulses every 30 s) with bipolar tungsten electrodes placed into area CA3 [27]. The EPSP initial slope (mV/ms) was determined from the average waveform of four consecutive responses. After determining the input/output relationship, long-term potentiation (LTP) was induced by a high-frequency stimulus (four trains of 100 Hz, 1 s stimulation separated by 5 min) with a success rate > 90%. Field EPSP initial slopes from averaged traces after LTP induction were normalized to baseline.

### Statistical analysis

Statistical analyses were performed using GraphPad Prism version 8.1 for Windows (GraphPad Software) using the unpaired one- or two-tailed Student’s t test and Regular or Row-Matched Two-way ANOVA test followed by Bonferroni’s multiple comparisons test. Data were considered significant when *P* values were < 0.05 (*), < 0.01(**) or < 0.001 (***).

## Results

### Analysis of AD brains reveals autophagy alterations and reduced NRBF2 expression in the parahippocampal gyrus and hippocampus

**Fig. 1:**
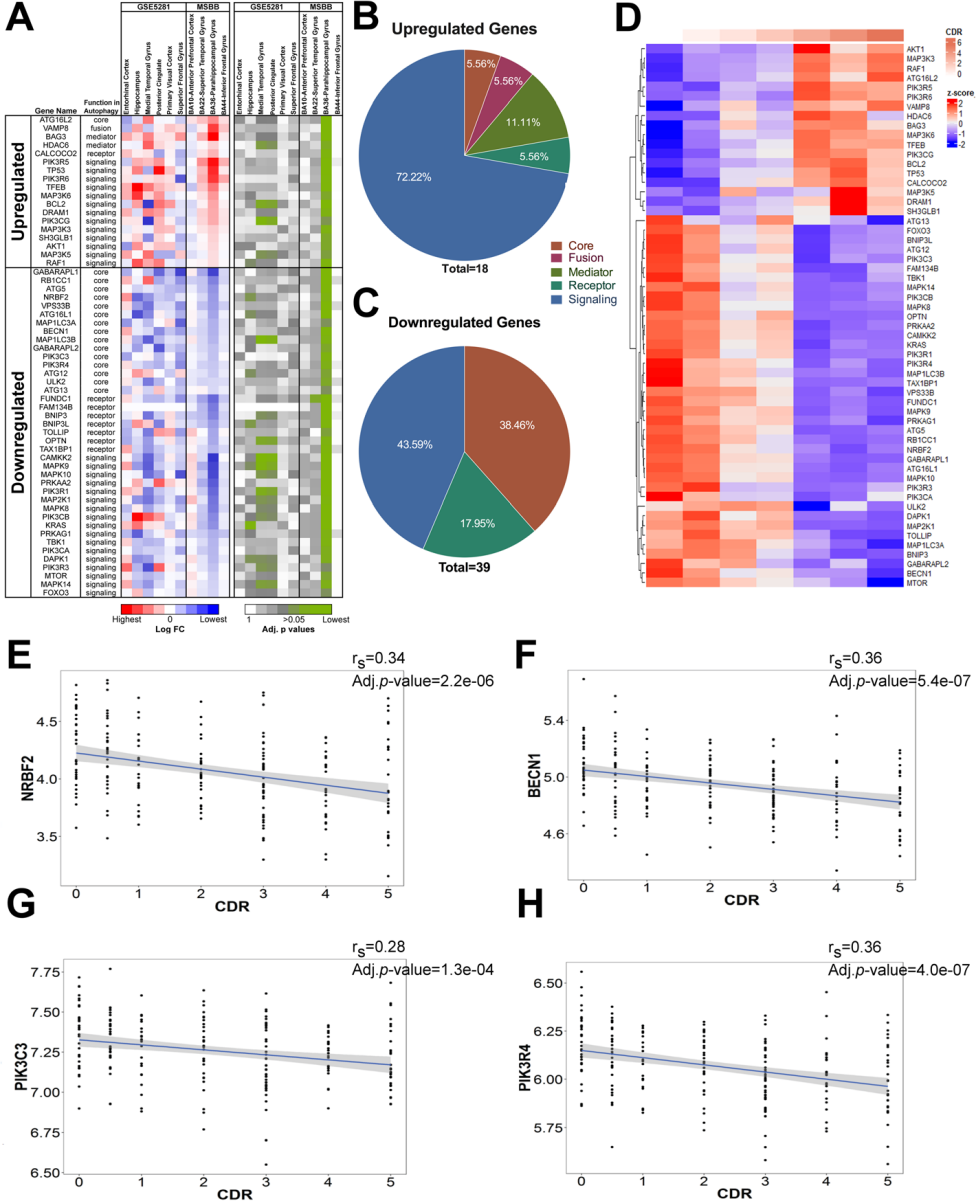
ATGs expression is altered in PHG and hippocampus of AD postmortem brains. **a** Heatmap of the Log FC and adjusted *p* values of the significantly up- and down-regulated ATG expressions found in PHG (BA36) and their counterpart expression in GSE5281 and other MSBB datasets. **b**-**c** Pie charts representing the autophagy gene functions associated to the upregulated or downregulated ATGs identified in (**a**). **d** Heatmap representing the mean z-score expression of each significant ATGs at different CDR stages within the PHG (MSBB-BA36). **e**-**h** *NRBF2*, *BECN1*, *PIK3C3* and *PIK3R4* gene expressions are progressively reduced in the PHG as a function of CDR score

We performed differentially expressed gene (DEG) analysis and examined the fold change of ~ 130 ATGs in multiple brain areas by analyzing transcriptomic datasets collected from late-onset AD (LOAD) postmortem brains (Mount Sinai Brain Bank, MSBB) [20] and laser-capture microdissected (LCM) neuron-enriched extracts from AD brains (GSE5281) [28] (Table 1 and Additional file 1: Table S1), thus greatly enhancing the statistical power of this study compared to others [11, 12, 16]. The DEG analysis of the MSBB cohort showed only one out of 4 brain regions, i.e. parahippocampal gyrus (PHG), with significant changes in more than 50 ATGs (Fig. 1a and Additional file 2: Figure S1A-B). The upregulated genes are functionally clustered as upstream autophagy regulators, while the downregulated genes are enriched for core autophagy machinery including autophagosome biogenesis, such as *GABARAPL1, ATG5, NRBF2, BECN1-PIK3C3* complex, *ULK1/2-ATG13-RB1CC1/FIP200* complex and a few signaling molecules of autophagy. Of note, downregulation of *NRBF2* and a few other proteins are also statistically significant in hippocampal neurons of AD brains, as well as in medial temporal gyrus and posterior cingulate, based on the GSE5281 data (Fig. 1a-c). Expression pattern of these 50 ATGs correlates either positively or negatively with the clinical dementia rating (CDR) score (Fig. 1d). Interestingly, *NRBF2, BECN1, PIK3C3* and *PIK3R4*, which encode different protein subunits of the same lipid kinase complex, are all significantly reduced, and their expression inversely correlate with the CDR score in the PHG (Fig. 1e-h). Given the functional relationship of these proteins in the PIK3C3 kinase complex, the above data suggest that a progressive decline of the NRBF2-associated BECN1-PIK3C3 kinase activity might occur during the course of AD in the PHG and hippocampus. In fact, we recently reported that NRBF2 and Beclin 1 expression were also reduced in hippocampus of 5XFAD mouse model [29]. Thus, we decided to further investigate the role of NRBF2 in AD related symptoms.

**Table 1:** Description of the dataset used in this study

Sample	Brain Bank (Template)	Brain Region	Control	AD
Tissue	MSBB (RNAseq)	BA10-Anterior prefrontal cortex	59	223
		BA22-Superior Temporal Gyrus		
		BA36-Parahippocampal Gyrus		
		BA44-Inferior Frontal Gyrus		
LCMed Neurons	GSE5281 (Array)	Entorhinal Cortex	13	10
		Hippocampus	13	10
		Medial Temproral Gyrus	12	16
		Posterior Cingulate	13	9
		Superior Frontal Gyrus	11	23
		Visual Cortex	12	19

### *NRBF2* depletion reduces autophagy, causes memory deficits, and impairs long-term potentiation

**Fig. 2:**
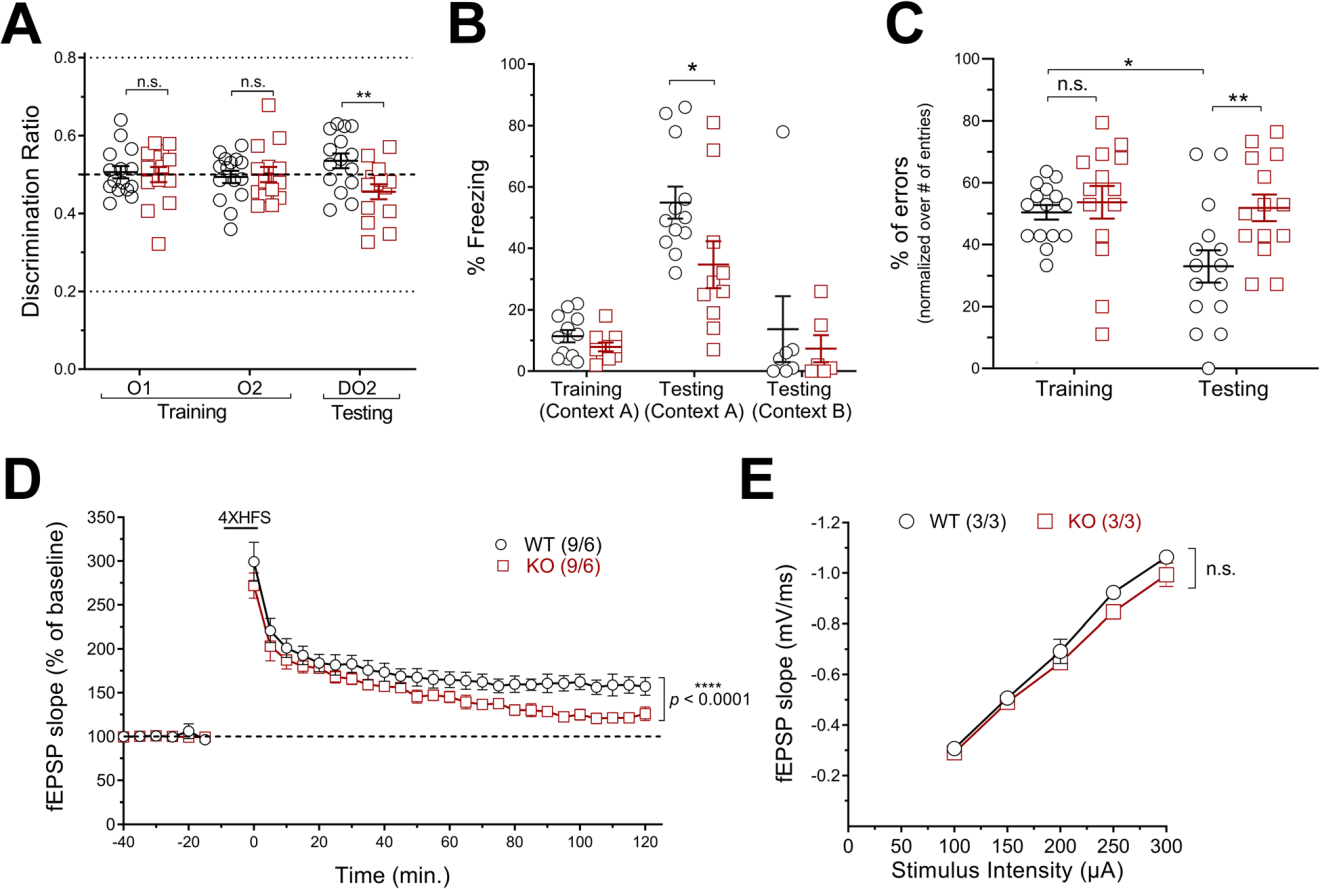
Loss of NRBF2 causes memory and LTP deficits in mice. **a** Discrimination ratio obtained from OLT task. Results are mean ± SEM of 3 months old WT (*n* = 15) and KO (*n* = 14) mice. **b** Freezing behavior recorded during CFC experiments. Results are mean ± SEM of 3 months old WT (*n* = 12) and KO (*n* = 10) mice. **c** Percentage of errors measured during RAM experiments. Results are mean ± SEM of3 months old WT (*n* = 15) and KO (*n* = 14) mice. The statistical significance was determined using row-matched two-way ANOVA test followed by Bonferroni’s post-test. **d** fEPSP slope measured from Schaffer collateral path. Results are mean ± SEM of 3 months old WT (*n* = 6) and KO (*n* = 6) slices from three different mice per group. The statistical significance was determined using regular two-way ANOVA on the last hour of recording. **e** Basal synaptic transmission measured from WT and KO mice. Results are mean ± SEM of 3 months old WT (*n* = 3) and KO (*n* = 3) slices from three different mice per group

Previous studies have shown the scaffolding role of NRBF2 in BECN1-PIK3C3 and ULK1 complexes assembly in cell lines [22, 30–34]. We have shown that *NRBF2* deletion reduces BECN1-PIK3C3-lipid kinase activity in the brain [22] and further demonstrated that ULK1-FIP200 complex stability is also compromised (Additional file 2: Figure S2A-D). Therefore, we evaluated the autophagy flux in different brain regions and AD-related phenotypes in these KO mice. First, we noticed a higher expression of *NRBF2* in the hippocampus and striatum compared to other brain regions of wild-type (WT) mice. Upon *NRBF2* deletion, autophagy markers, p62 and LC3-II, accumulated most significantly in the hippocampus (Additional file 2: Figure S3A-D, F) [35]. We observed little change in p62 and LC3A/B mRNA levels in *NRBF2-KO* hippocampus (Additional file 2: Figure S3E), suggesting that p62 or LC3A/B protein increase is caused by autophagy impairment rather than enhancement of their gene expression. We next performed multiple behavioral tasks to test hippocampal-associated functions. Using open field (OF), light-dark (LD) box and elevated-plus maze (EPM) tasks, we observed that anxiety-related behavior was unchanged in young *NRBF2-*KO animals (Additional file 2: Figure S4A-C), suggesting that ventral hippocampal functions are unaffected in these mice [36]. Second, we performed an object-location task (OLT) to specifically assess spatial memory, known to rely on proper dorsal hippocampus function [37]. After habituation and training, we found that *NRBF2-*KO mice performed poorly in discriminating the new location of a familiar object, thus suggesting spatial memory deficits in the mutant mice (Fig. 2a). Third, we performed contextual fear conditioning (CFC) test. On the testing day, we observed that the freezing behavior of *NRBF2-*KO mice in the training-context A is reduced when compared to the WT littermates, while no significant change in observed in the context B, thus supporting that loss of *NRBF2* causes memory deficits (Fig. 2b). Fourth, we performed a radial-arm maze (RAM) test. *NRBF2*-KO mice showed an impairment in remembering previously visited arm, as revealed with an increase percentage of errors made on the testing day when compared to the littermate controls (Fig. 2c). We also observed that loss of NRBF2 expression in 5XFAD mice enhances their memory deficits using OL, CFC and RAM behavioral task (Additional file 2: Figure S5A-C).

To further understand the basis of the memory deficits in *NRBF2-*KO mice, we examine long-term potentiation (LTP), a well-known cellular mechanism linked to learning and memory. Results of the field recordings from the Schaffer collateral pathway showed a reduced maintenance of LTP in *NRBF2-*KO animals when compared to WT (Fig. 2d), while no change in basal synaptic transmission (Fig. 2e) or expression of pre- and post-synaptic markers (Additional file 2: Figure S6A-C) were observed. Given that AMPK signaling modulates autophagy [38], memory and LTP processes [14, 39, 40], we decided to examine the phosphorylation of AMPK-Thr172 as well as the phosphorylation of Raptor-Ser792 — an AMPK substrate [41] — to understand further the molecular changes occurring in *NRBF2*-KO hippocampus that could relate to LTP deficit. Western blot analysis showed higher phosphorylation levels of AMPK-Thr172 and Raptor-Ser792 in *NRBF2*-KO animals, whereas no change of their total protein levels was detected (Additional file 2: Figure S7A-C). We also performed immunofluorescence imaging experiments and showed that CA1 and CA3 pyramidal neurons of *NRBF2*-KO mice had enhanced phospho-AMPK-T172 signal, thus validating the hyperactivation of AMPK in *NRBF2-KO* hippocampus (Additional file 2: Figure S7D-E). While increased phosphorylation of Raptor-Ser792 is known to inhibit mTOR kinase function, we examined mTOR catalytic activity through assessment of its autophosphorylation site, i.e. Ser2481, [42] and phosphorylation of 4E-BP1-Thr37/46 [43], a known mTOR substrate, while both are crucial for LTP and memory consolidation [44–46]. Immunoblot analysis demonstrated that phosphorylation of mTOR-Ser2481 and 4E-BP1-Thr37/46 were decreased in hippocampal extracts of *NRBF2*-KO mice when compared to the littermate controls, while no change in these proteins basal expression was observed (Additional file 2: Figure S7F-H). Together, these data suggest that hyperactivation of AMPK and reduced mTOR activity could be related to LTP and memory deficits in *NRBF2-KO* mice.

### Loss of *NRBF2* promotes accumulation of APP C-terminal fragments and Aβ in mouse hippocampus

**Fig. 3:**
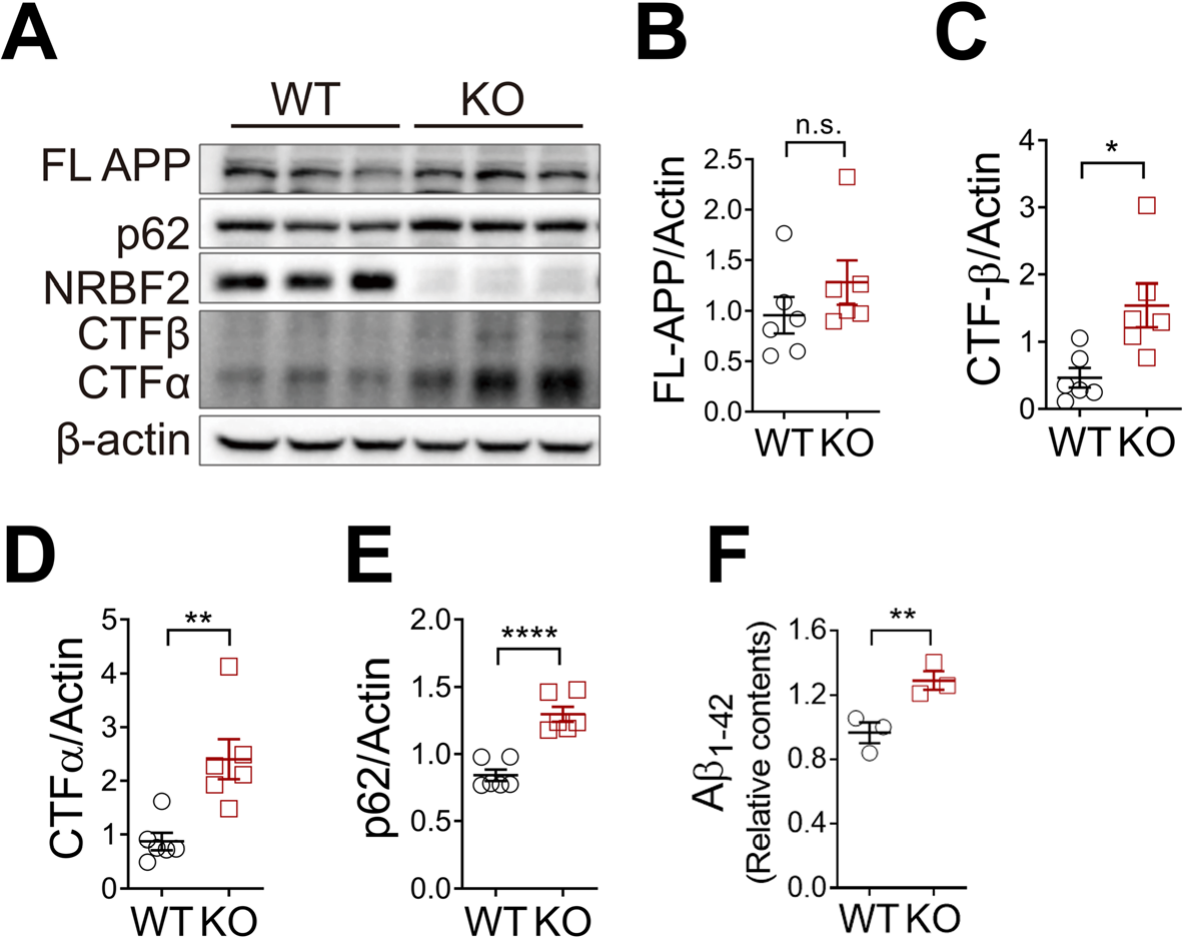
Deletion of NRBF2 enhances accumulation of APP-CTFs and Aβ_42_ in mouse hippocampus. **a** Expression of FL-APP, APP-CTFs in the hippocampus of 20-months-old WT (*n* = 6) and *NRBF2*-KO (*n* = 6) mice. The blots shown are representative of two separate experiments (**b**-**e**) Quantification of (**a**). The statistical significance was determined using two-tailed unpaired Student’s t-test. **f** ELISA analysis results of Aβ_1–42_ levels in hippocampus of 20- month-old WT (*n* = 3) and *NRBF2*-KO (*n* = 3) mice. The statistical significance was determined using two-tailed unpaired Student’s t-test. *p < 0.05, **p < 0.01, ****p < 0.0001

We next evaluated the Aβ level in the hippocampus of aged *NRBF2-*KO mice. We detected an accumulation of p62 and APP Carboxyl-terminal Fragments (APP-CTFs) in late adult hippocampus of *NRBF2*-KO mice while observing no significant change in full-length APP (FL-APP) when compared to control (Fig. 3a-e). Our study revealed an increase of Aβ_1–42_ levels in aged KO hippocampal tissue (Fig. 3f). To verify that *NRBF2* deletion also promotes the buildup of human insoluble Aβ_42_, we crossed *NRBF2-*KO mice with 5XFAD mouse model — a well characterized AD model known to overproduce Aβ_42_ [47] — and found that *NRBF2-*KO mice carrying 5XFAD hemizygous allele contained enhanced levels of insoluble Aβ_42_ in the hippocampus compared to controls (Additional file 2: Figure S5D), further supporting that *NRBF2* deficiency accelerates the aggregation of insoluble Aβ_42_ in the hippocampus.

### AAV transduction of *NRBF2* into the dorsal hippocampus rescues autophagy and memory impairments of *NRBF2-KO* and reduces β-amyloid load in 5XFAD mice

**Fig. 4:**
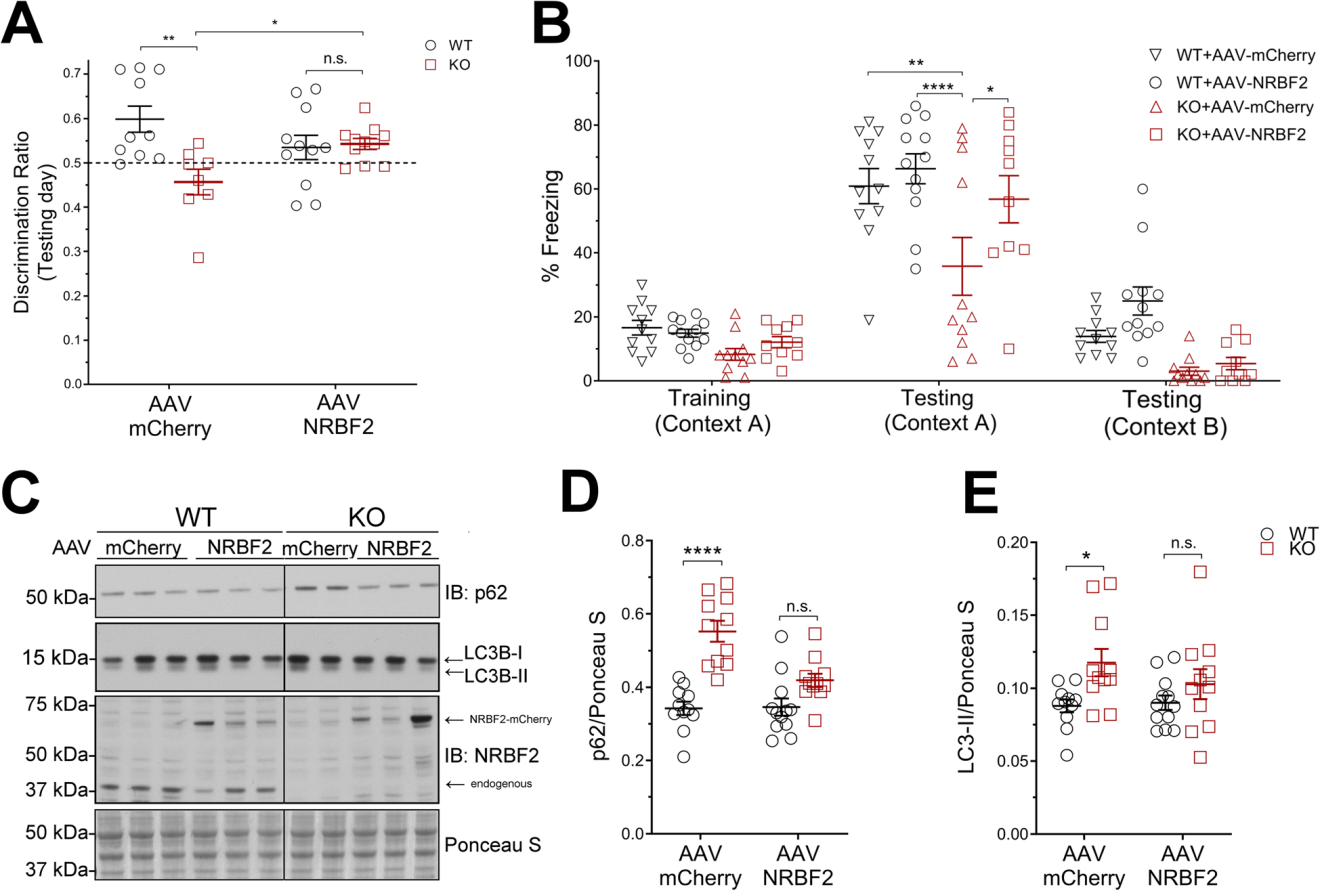
Transduction of NRBF2 carrying viruses into dorsal hippocampus rescues memory impairments and restores autophagy in *NRBF2*-KO mice. **a** Discrimination ratio obtained on testing day of OLT. **b** Freezing behavior recorded during CFC experiments. Results are mean ± SEM of 3–4 months old WT + mCherry (*n* = 14), WT + NRBF2 (*n* = 14), KO + mCherry (*n* = 16) and KO + NRBF2 (*n* = 18) mice. **c** Immunoblot (IB) analysis of p62 and LC3B. **d**-**e** Quantification of C. Results are mean ± SEM of 3–4 months old WT + mCherry (*n* = 11) and WT + NRBF2 (*n* = 12) KO + mCherry (*n* = 11) KO + NRBF2 (*n* = 11) mice. The statistical significance was determined using regular two-way ANOVA test followed by Bonferroni’s post-test. *p < 0.05, **p < 0.01, ***p < 0.001, ****p < 0.0001

To test the specific function of hippocampal *NRBF2* in maintaining memory integrity, we reinstated *NRBF2* expression in the hippocampal areas of *NRBF2*-KO mice by injecting recombinant adeno-associated viruses (rAAV) carrying either mCherry (AAV9-CMV-mCherry) or NRBF2-mCherry (AAV9-CMV-NRBF2-mCherry) into the dorsal hippocampus (dHip) of 2–3 months old WT and *NRBF2-KO* mice. We then performed behavioral and biochemical analyses of these injected mice at 21–30 days post-injection. Through assessment of OLT and CFC tasks, we found that NRBF2-mCherry-injected KO mice showed greater discrimination and improved freezing behaviors when compared to mCherry-injected KO mice; and no differences was detected between mCherry-injected and NRBF2-mCherry injected WT mice (Fig. 4a-b). The above results demonstrate that the memory deficits in *NRBF2*-KO mice is caused by *NRBF2* loss of function specifically in the dorsal hippocampus and can be reversed by reintroduction of NRBF2 expression. Further, our data suggests that the memory deficit of *NRBF2*-KO mice is unlikely caused by developmental effect or neurodegeneration. Indeed, we have not detected any signs of apoptosis in *NRBF2-KO* mouse hippocampus (Additional file 2: Figure S8A-B). Next, we examined autophagy status and found comparable levels of p62 or LC3-II proteins between NRBF2-mCherry-injected WT and KO mice, whereas p62 or LC3-II levels remained different between mCherry-injected WT and KO mice (Fig. 4c-e), suggesting a recovery of autophagy in NRBF2-mCherry-injected KO mice. Moreover, ULK1-FIP200 interaction was improved in NRBF2-mCherry-injected KO mice when compared to mCherry-injected KO mice (Additional file 2: Figure S2E-F).

**Fig. 5:**
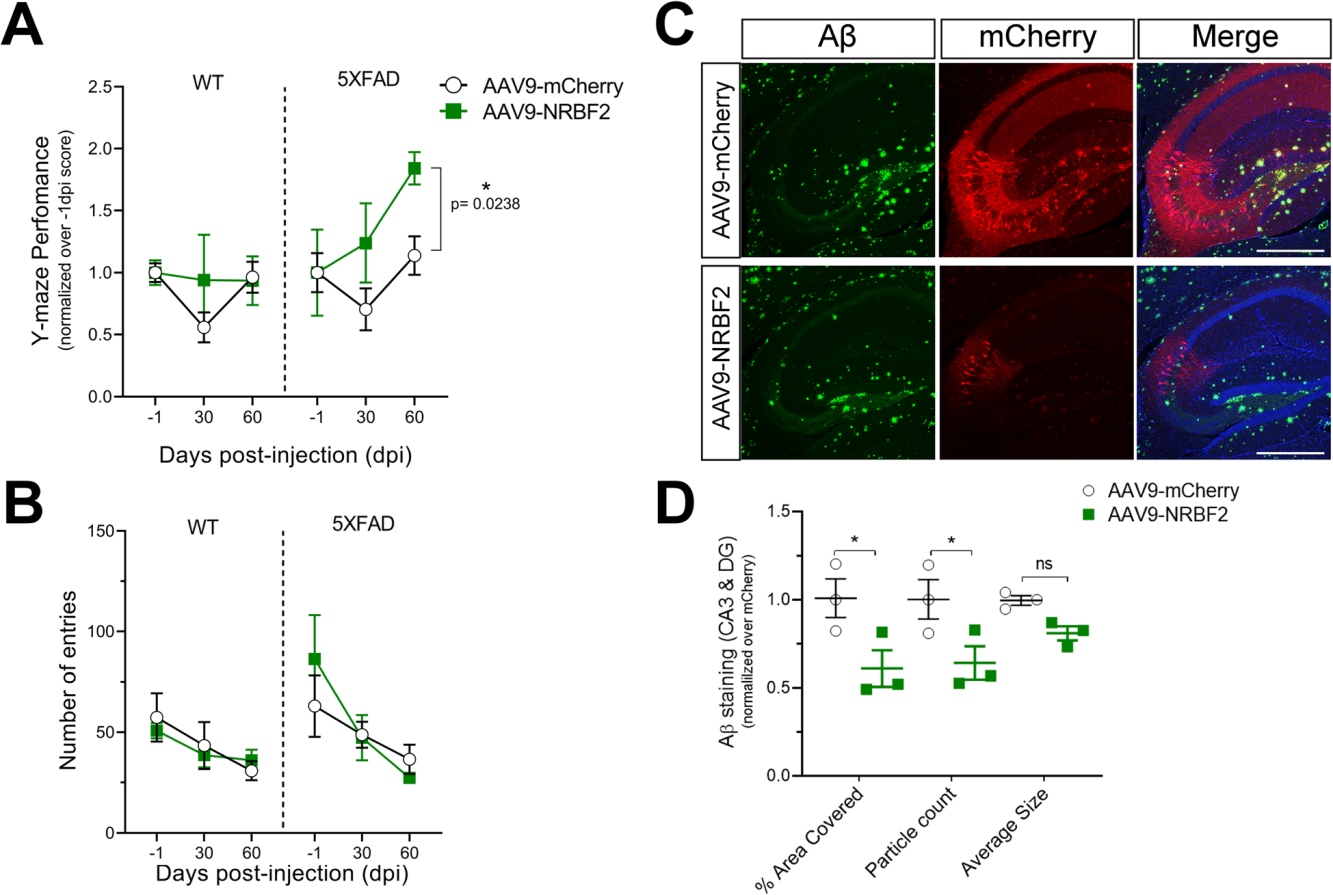
Injection of rAAV-NRBF2-mCherry into dorsal hippocampus rescues memory impairments and reduces Aβ levels in 5XFAD mice. **a**-**b** Y-Maze performance and total number of entries recorded 1 day before (− 1), 30 and 60 days post-viral injection. Each group performance was normalized to their initial performance, i.e. -1 dpi data. Results are mean ± SEM of 4–5 months old WT + mCherry (*n* = 4), WT + NRBF2 (*n* = 4), 5XFAD + mCherry (*n* = 4), 5XFAD + NRBF2 (*n* = 4) mice. The statistical significance was determined using a repeated two-way ANOVA test. **c** Immunofluorescence analysis of Aβ and mCherry expression in hippocampus of 5XFAD mice injected with mCherry or NRBF2-mCherry viruses. Scale bars, 500 μm. Images shown are representatives of two different experiments. **d** Quantification of Aβ signal within CA3 and DG area C. Results were normalized over mCherry control and are mean ± SEM of mCherry or NRBF2-mCherry acquired from three separate mice per group. The statistical significance was determined using regular two-way ANOVA test followed by Bonferroni’s post-test. *p < 0.05

Finally, we injected rAAV carrying *NRBF2* into dHip of 5XFAD mice and tested their cognitive functions before and at 30- and 60-days post-injection (dpi). To do so we chose to use a spontaneous alternation task (SAT) since it is non-invasive and easily repeatable when compared to OLT and CFC tasks. We found that the SAT performance of NRBF2-mCherry-injected 5XFAD mice was improved when compared to that of mCherry-injected 5XFAD mice, while no change was observed between NRBF2-mCherry- and mCherry-injected WT mice (Fig. 5a). Of note, the total numbers of entries showed no significant differences between the groups (Fig. 5b). Importantly, while evaluating β-amyloid load in the injected 5XFAD brains, we observed a reduction of area coverage and the counts of particles as well as a trend decrease of the particles average size, of Aβ staining in NRBF2-mCherry-injected mice at 60 dpi, when compared to mCherry-injected littermate controls, despite lower expression of NRBF2-mCherry than mCherry (Fig. 5c-d), suggesting that overexpression of *NRBF2* decreases β-amyloid level in the hippocampus. Taken together, our data also suggest that increasing NRBF2 expression is neuroprotective as evidenced by lowering β-amyloid load and improving memory function in AD model.

## Discussion

Several studies have attempted to characterize the changes of autophagy genes in AD brains. However, the results lack an agreement on the exact nature of the change in autophagy. By leveraging the transcriptomic dataset of a large AD cohort, our analysis reveals altered expression of specific functional groups of autophagy genes in the PHG of AD patients. Our data shows that downregulation of various autophagy kinase complex components, e.g. *BECN1-PIK3C3*, *ULK1/2-ATG13-FIP200*, and *NRBF2,* is prominent and coincides with the disease progression (CDR). Indeed, dysregulation of *Beclin 1-PIK3C3* and *ULK1* have already been reported in AD brains [10, 11, 13, 14, 48]. However, the significance of *NRBF2* or its cellular functions in learning and memory has never been demonstrated.

Our study is the first to demonstrate that loss of *NRBF2* and NRBF2-associated protein complex integrities promote memory impairments in young animals. Our findings of reduced expression of *NRBF2* (along with other ATGs) in AD brains and characterization of *NRBF2-KO* mice support the role of impaired NRBF2-associated function in promoting memory dysfunctions and AD risk. Specifically, our study demonstrates that *NRBF2*-KO mice develop memory deficits through multiple cognition assays, i.e. working memory (RAM), reference memory (CFC) and recognition memory (OLT), while displaying minor change in anxiety-related behavior based on OF, LD and EPM studies. Thus, our data may suggest selective vulnerability of hippocampal regions responsible for memory function caused by deletion of *NRBF2*. Interestingly, these cognitive domains are known to be impaired in different AD mouse models and are recognized to model the preclinical behavioral changes observed in AD [49]. Our work therefore provide insight into how autophagy related processes, mediated by NRBF2, could potentially modulate pathogenic pathways in AD.

Furthermore, we showed that depletion of *NRBF2* alters ULK1-FIP200 complex, in addition to Beclin 1-PIK3C3 complex as we previously reported [22]. Reestablishing NRBF2 expression by viral transduction into dorsal hippocampus of KO mice rescues memory impairment, autophagy flux, ULK1-FIP200 interaction, thus supporting that memory deficits are unlikely caused by developmental effect or neurodegeneration. Therefore, the lack of NRBF2-related functions in the hippocampus primarily accounts for the memory impairment in the mutant mice. Our work shows an impairment of LTP while observing no change in basal synaptic transmission in the hippocampus of *NRBF2*-KO mice. However, the precise mechanism that contributes to LTP disruption remains to be clarified. One could suspect that a modification in AMPA receptors (AMPAR) trafficking is causing the LTP impairment or cognitive deficits in the mutant mice. In fact, BECN1-PIK3C3 and ULK1/2 complexes have been shown to promote endocytosis and ER-to-Golgi trafficking respectively [50, 51]. Therefore, dysregulation of the functions of these kinases beyond autophagy in the hippocampus of *NRBF2-KO* mice could disrupt the AMPAR trafficking and explain the LTP inhibition. Nevertheless, deeper mechanistic analysis is required to precisely define the molecular components contributing to the LTP impairment observed in *NRBF2-KO* mice.

## Conclusions

In summary, our findings identify progressive decline in the expression of *NRBF2* and *NRBF2*-associated autophagy complex in specific brain regions of AD patients, which correlates with clinical dementia progression. Our investigation reveals the impact of dysfunctional *NRBF2*-related pathways in promoting Aβ accumulation and memory deficits in experimental animal models. Our study also provides evidence that restoration or modulation of NRBF2 and perhaps its associated kinase complexes activities may represent a new therapeutic strategy for improving memory impairment related to AD.

## Supplementary information

**Additional file 1: Table S1.** T-stats* and adjusted p-values.

**Additional file 2: Figure S1.** Autophagy gene expression in multiple region of AD brains. **Figure S2.** Loss of NRBF2 reduces ULK1-FIP200 interaction and is rescued upon NRBF2-viruses transduction. **Figure S3.** Brain regional analysis of NRBF2 and autophagy markers expression. **Figure S4.** No anxious or anxiolytic behavior is observed in NRBF2-KO mice. **Figure S5.** Loss of NRBF2 in 5XFAD mice promotes memory deficits and enhances Aβ aggregation. **Figure S6.** Pre- and post-synaptic markers expression is unchanged in NRBF2-KO hippocampi. **Figure S7.** Upregulation of AMPK activity inhibits mTORC1 signaling in hippocampus of NRBF2-KO mice. **Figure S8.** Level of cleaved-caspase 3 is not changed in hippocampus of *NRBF2* KO mice.

## Data Availability

The datasets supporting the conclusions of this article are included within the supplementary information available at *Molecular Neurodegeneration* website.

## Abbreviations

AAV: Adeno-associated virus
AD: Alzheimer’s Disease
AMPK: AMP-activated protein kinase
ATG: Autophagy-related
ATG13: Autophagy-related protein 13
Aβ: amyloid-β
BECN1: Beclin 1
CFC: Contextual fear conditioning
ELISA: Enzyme-linked immunosorbent assay
FIP200: FAK family kinase-interacting protein of 200 kDa
KO: Knockout
LC3: Microtubule-associated proteins 1A/1B light chain 3
LC3-II: LC3-phosphatidylethanolamine conjugate
LTP: Long-Term Potentiation
mTOR: Mammalian Target of Rapamycin
NRBF2: Nuclear Receptor Binding Factor 2
OLT: Object location task
p62/SQSTM1: Sequestosome-1
PIK3C3: Class III PI3-kinase
pTau: Hyperphosphorylated-tau
RAM: Radial-arm maze
SAT: Spontaneous alternation task
ULK1: Unc-51 Like Autophagy Activating Kinase 1
WT: Wild type
