# Supplementary material for: Autophagy protein NRBF2 has reduced expression in Alzheimer’s brains and modulates memory and amyloid-beta homeostasis in mice
Source: Mol Neurodegener. 2019 Nov 27;14:43. doi: 10.1186/s13024-019-0342-4 (PMC6882183; doi:10.1186/s13024-019-0342-4)
Supplement: Supplementary file 2 — Additional file 2: Figure S1. Autophagy gene expression in multiple region of AD brains. Figure S2. Loss of NRBF2 reduces ULK1-FIP200 interaction and is rescued upon NRBF2-viruses transduction. Figure S3. Brain regional analysis of NRBF2 and autophagy markers expression. Figure S4. No anxious or anxiolytic behavior is observed in NRBF2-KO mice. Figure S5. Loss of NRBF2 in 5XFAD mice promotes memory deficits and enhances Aβ aggregation. Figure S6. Pre- and post-synaptic markers expression is unchanged in NRBF2-KO hippocampi. Figure S7. Upregulation of AMPK activity inhibits mTORC1 signaling in hippocampus of NRBF2-KO mice. Figure S8. Level of cleaved-caspase 3 is not changed in hippocampus of NRBF2 KO mice. [file 13024_2019_342_MOESM2_ESM.docx]

**SUPPLEMENTARY INFORMATION**

**AUTOPHAgY PROTEIN NRBF2 SHOWS REDUCTION IN ALZHEIMER'S BRAINs AND MODULATES MEMORY AND Amyloid-Beta homeostasis IN MICE**

**AUTHORS**

Véronik Lachance^1^, Qian Wang^1, 2^, Eric Sweet^1, 3, 4^, Insup Choi^1^, Cui-Zan Cai^5^, Xu-Xu Zhuang^5^, Yuanxi Zhang^1^, Jessica Li Jiang^1^, Robert D. Blitzer^3^, Ozlem Bozdagi-Gunal^6, 7^, Bin Zhang^2^, Jia-Hong Lu^5, 8^, and Zhenyu Yue^1, 8^.

**AFFILIATIONS**

^1^ Department of Neurology, The Friedman Brain Institute, Icahn School of Medicine at Mount Sinai, New York, NY 10029, USA, ^2^ Department of Genomics and Genetic Sciences, Icahn School of Medicine at Mount Sinai, New York, NY 10029, USA, ^3^ Departments of Psychiatry and Pharmacological Sciences, Icahn School of Medicine at Mount Sinai, New York, NY 10029, USA, ^4^ Current address: Department of Biology, West Chester University, West Chester, PA 19383, USA, ^5^ State Key Laboratory of Quality Research in Chinese Medicine, Institute of Chinese Medical Sciences, University of Macau, Taipa, Macau SAR, China, ^6^ Department of Neuroscience, Icahn School of Medicine at Mount Sinai, New York, NY 10029, USA, ^7^ Current address: Department of Psychiatry, Rutgers New Jersey Medical School, Newark, NJ 07103, USA, ^8^ Correspondence should be addressed to Z.Y. (e-mail: [zhenyu.yue@mssm.edu](file:///C:\Users\sinde\AppData\Local\Packages\Microsoft.MicrosoftEdge_8wekyb3d8bbwe\TempState\Downloads\zhenyu.yue@mssm.edu)) and J-H.L. (email: [jiahonglu@um.edu.mo](mailto:jiahonglu@um.edu.mo))

**SUPPLEMENTAL METHODS**

**Triton-X-100 soluble and insoluble Fractionation Assay**

Hippocampal tissue was homogenized and disrupted in PBS containing 1% Triton X-100 and phosphatase/protease inhibitors and incubated on ice for 30 min. After centrifugation at 15,000 g for 30 min at 4 **°**C, Triton X-100 soluble fractions were collected. Pellets were washed 4x with 200 μl of the buffer mentioned above. Pellets were further solubilized in PBS containing 1% SDS, 1% Triton X-100, and phosphatase/protease inhibitors and incubated in 60 **°**C water bath for 1 h. Triton X-100 insoluble fractions were collected after centrifugation at 15,000 g for 30 min at 4 **°**C. BCA assay was done on the extracts to ensure equal protein concentrations. This procedure was described elsewhere [1, 2].

**Human Aβ_42_ ELISA**

Triton-X-100 soluble and insoluble Aβ_42_ level was quantify from hippocampal extracts. Fractions were analyzed in duplicate. Same protein amount was loaded into each well, and the plate was incubated overnight at 4°C with gentle agitation. ELISA was performed according to the manufacturer’s instructions (#KHB3441, Thermo Scientific).

**Quantitative PCR**

Total mRNA was isolated from hippocampal homogenates of 3-4 months old male mice using QIAGEN RNeasy kits (#74104) and following the manufacturer’s instructions. Quantitative PCR (qPCR) was performed with ABI PRISM 7900HT Detection System (Applied Biosystems; ThermoFisher Scientific) using SYBR Green reagents. Analysis was done by using the 2^-ΔCt^ method described in [3]. Primers for transcripts analyzed were as follows: p62-For: 5’-GAA GCT GCC CTA TAC CCA CA-3’; p62-Rev: 5’-GCC TTC ATC CGA GAA ACC CAT-3’; LC3a-For: 5’-CAA CTC AAC CCC ACG CAG G-3’; LC3a-Rev: 5’-CCT CTT GAC TCA GAA GCC GAA-3’; LC3b-For: 5’-CTT GCA GCT CAA TGC TAA CCA; LC3b-Rev: 5’-CTC ACT CTC GTA CAC TTC GGA-3’; p62-For: 5’-GAA GCT GCC CTA TAC CCA CA-3’; p62-Rev: 5’-GCC TTC ATC CGA GAA ACC CAT-3’; LC3a-For: 5’-CAA CTC AAC CCC ACG CAG G-3’; LC3a-Rev: 5’-CCT CTT GAC TCA GAA GCC GAA-3’; LC3b-For: 5’-CTT GCA GCT CAA TGC TAA CCA; LC3b-Rev: 5’-CTC ACT CTC GTA CAC TTC GGA-3’

**Behavior Studies**

*Open-Field (OF)*

The apparatus consisted of a clear Plexiglas box (40 × 40 cm, 40 cm high) from Omnitech Electronic, Inc. (Columbus, OH, USA). Animals were introduced into one corner of the test chamber and allowed free exploration for 60 min. individually under standard illumination conditions. Time spent in the central and peripheral areas were recorded by Fusion Software and plotted in 5 min. intervals.

*Light-Dark Box (LD)*

A black plastic box (20 cm L × 20 cm W × 40 cm H), was introduced into the open-field apparatus described above, dividing the arena into dark and light components. The box was equipped with a black plastic lid and had an opening on the bottom (5 cm diameter) to allow the mouse to enter the surrounding arena. Mice were placed into the black box, the lid was closed, and mice could move freely in the entire apparatus for 30 min. Time spent in light and dark compartments was recorded by Fusion Software. A decrease in time spent in the lit area reflects a state of increased anxiety, whereas an increase in time spent in this same zone reflects an anxiolytic behavior.

*Elevated-Plus Maze (EPM)*

The EPM (Med Associates) consisted of a center square (6 × 6 cm^2^), two open arms, and two closed arms (measuring 35 × 6 cm^2^ each). Black polypropylene walls measuring 20 cm in height enclosed the closed arms. Mice were placed in the center square facing one of the closed arms and were free to explore for 10 min. Time spent in each arm and the number of entries was recorded and scored by the EthoVision video tracking system (Noldus, Wageningen, The Netherlands). Animals that fell/jumped off the maze were removed from the study. All procedure described above were meticulously described in [4]. A decrease in time spent in the open arms reflected a state of increased anxiety, whereas an increase in time spent in these arms reflects an anxiolytic behavior.

**SUPPLEMENTARY FIGURES**

**B**

**
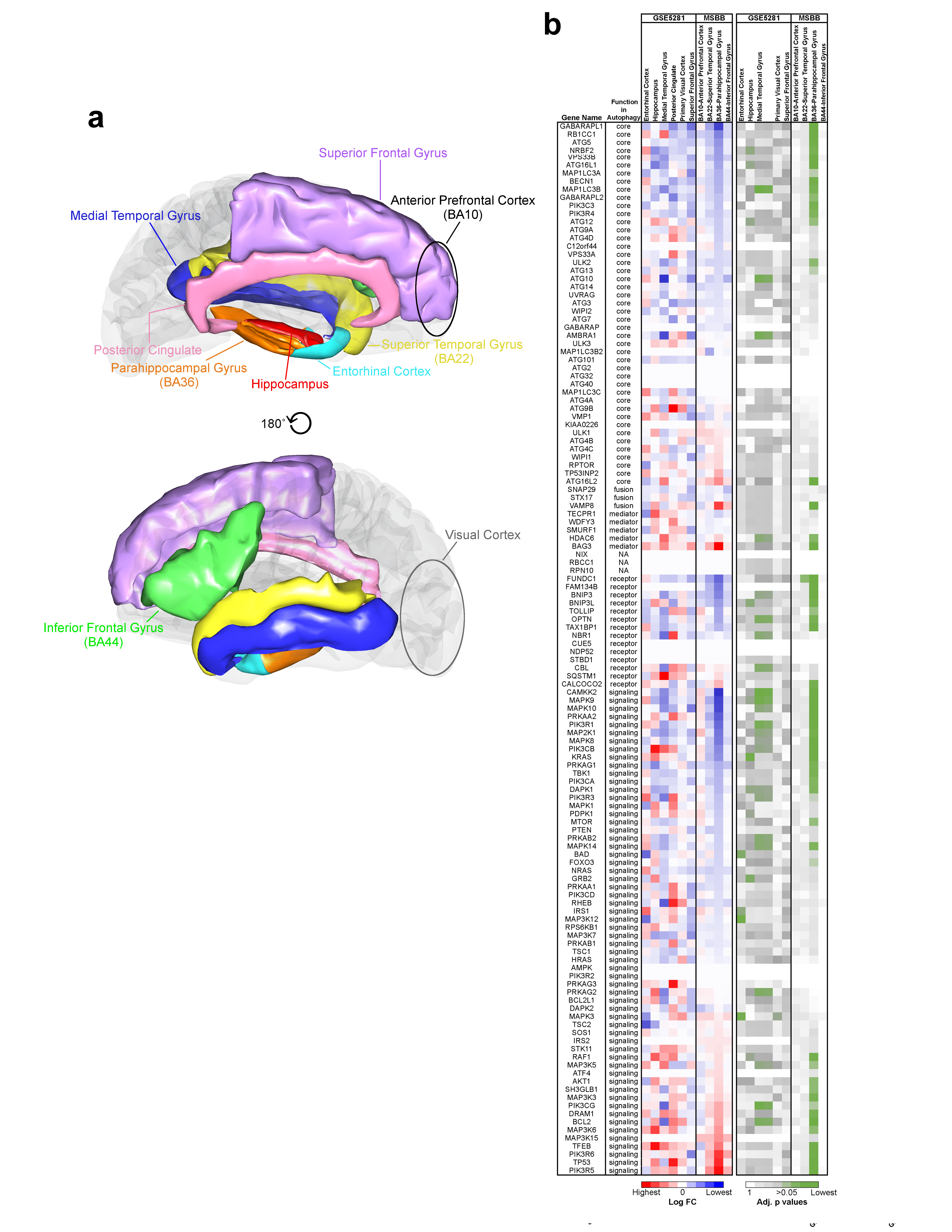
**

**A**

**Figure S1: Autophagy gene expression in multiple region of AD brains. (A)** Scheme of the different brain areas investigated during the study. Images produce with BodyParts3D website (<http://lifesciencedb.jp/bp3d/>). **(B)** Heatmap representing Log FC and adjusted p values of autophagy gene expression data amongst the different brain regions analyzed in the study.

**
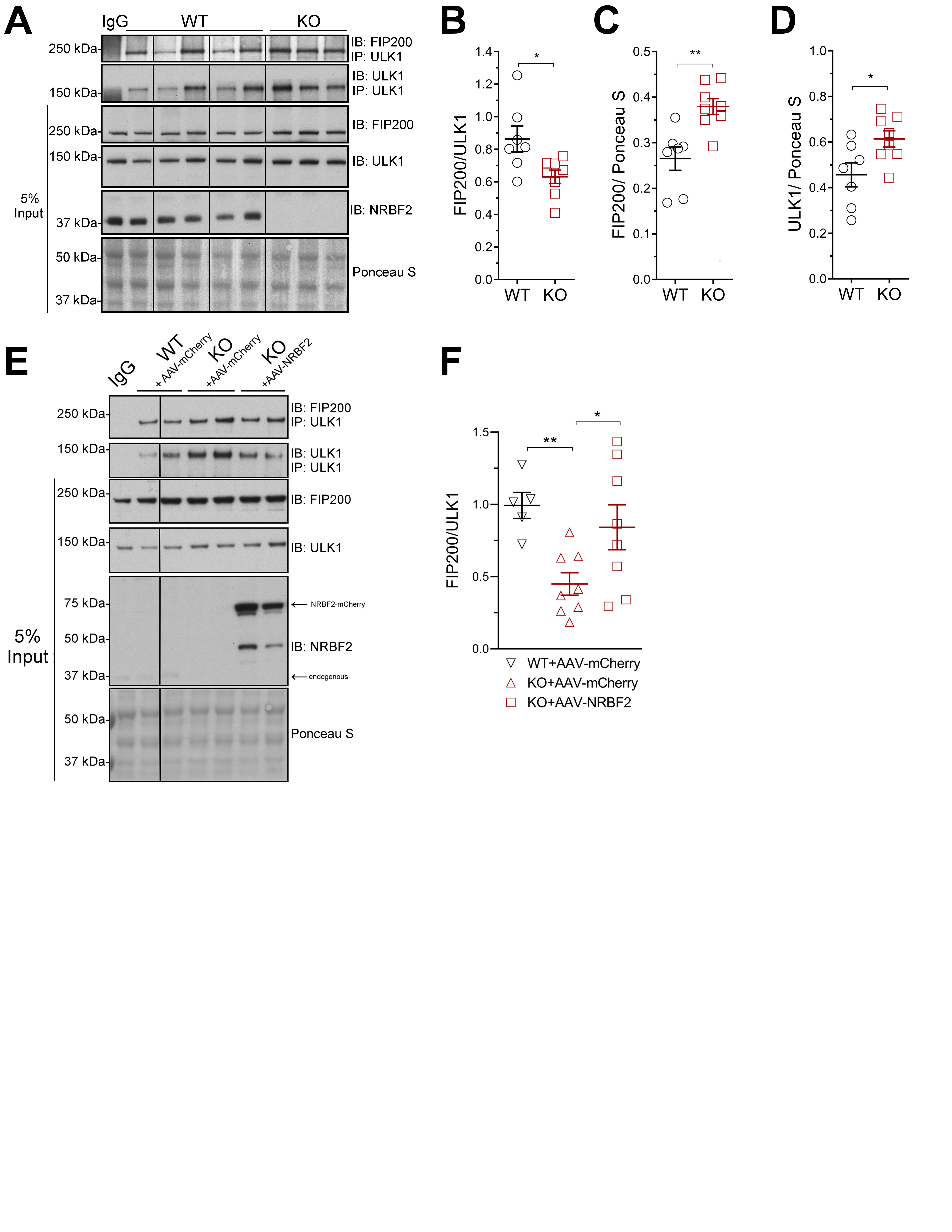
**

**Figure S2: Loss of NRBF2 reduces ULK1-FIP200 interaction and is rescued upon NRBF2-viruses transduction. (A)** Interaction analysis of ULK1 and FIP200 in WT and KO hippocampal homogenates. Immunoprecipitation (IP) has been performed with specific anti-ULK1 antibody and followed by Immunoblot (IB). The blots shown are representative of two separate experiments. Samples were run on the same gel but were noncontiguous. (**B-D)** Quantification of **A**. Results are mean±SEM of WT (n=7) and KO (n=7) mice. The statistical significance was determined using two-tailed unpaired Student's t-test, *p<0.05, **p<0.01. **(E)** Immunoprecipitation (IP) has been performed with specific anti-ULK1 antibody and followed by IB. **(F)** Quantification of **E**. Results are mean±SEM of WT+mCherry (n=5), KO+mCherry (n=8) and KO+NRBF2 (n=8) mice. The statistical significance was determined using one-way ANOVA test followed by uncorrected Fisher’s LSD post-test. *p<0.05, **p<0.01. The blots shown are representative of two separate experiments. Samples were run on the same gel but were noncontiguous.

**
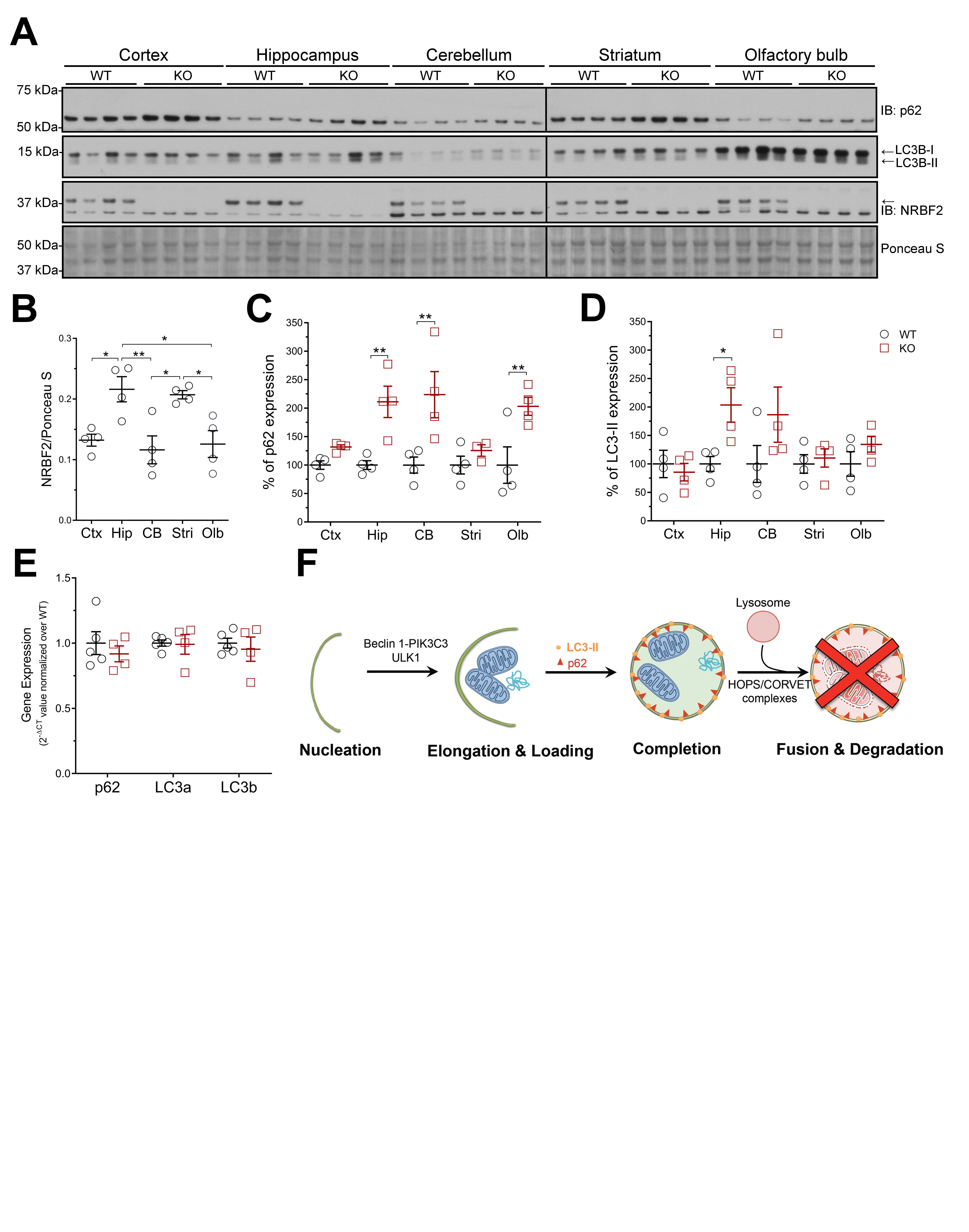
**

**Figure S3: Brain regional analysis of NRBF2 and autophagy markers expression. (A)** Western blot analysis of p62, LC3B and NRBF2 expression. The blots shown are representative of two experiments. **(B-D)** Quantification of **A**. Results are mean±SEM of WT (n=4) and KO (n=4) mice. The statistical significance was determined using one-way ANOVA or regular two-way ANOVA followed by Bonferroni’s post-test, *p<0.05, **p<0.01. **(E)** Gene Expression analysis of p62, LC3a and LC3b in NRBF2-WT and KO hippocampal tissue. Results are mean±SEM of WT (n=5) and KO (n=4) mice. The statistical significance was determined using two-way ANOVA test followed by Bonferroni’s post-test. **(F)** Representative scheme showing autophagy impairment.

**
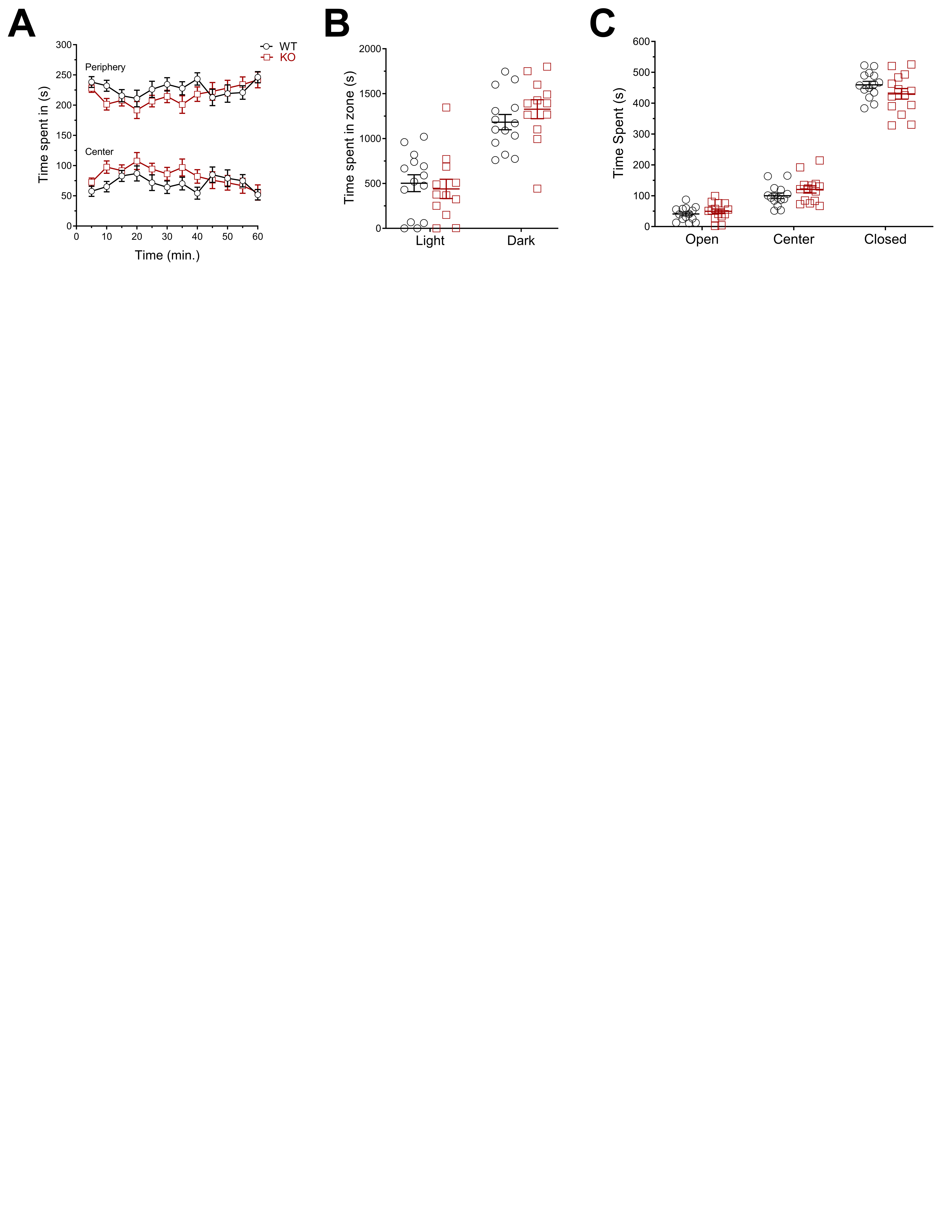
Figure S4: No anxious or anxiolytic behavior is observed in NRBF2-KO mice. (A)** Time spent in center and peripheral zone during open field analysis. Results are mean±SEM of WT (n=10) and KO (n=12) mice. The statistical significance was determined using repeated two-way ANOVA test. **(B)** Time spent in light or dark zone during light-dark box task. Results are mean±SEM of WT (n=10) and KO (n=12) mice. The statistical significance was determined using regular two-way ANOVA test followed by Bonferroni’s post-test. **(C)** Time spent in indicated zone during elevated-plus maze experiments. Results are mean±SEM of WT (n=10) and KO (n=12) mice. The statistical significance was determined using regular two-way ANOVA test followed by Bonferroni’s post-test.


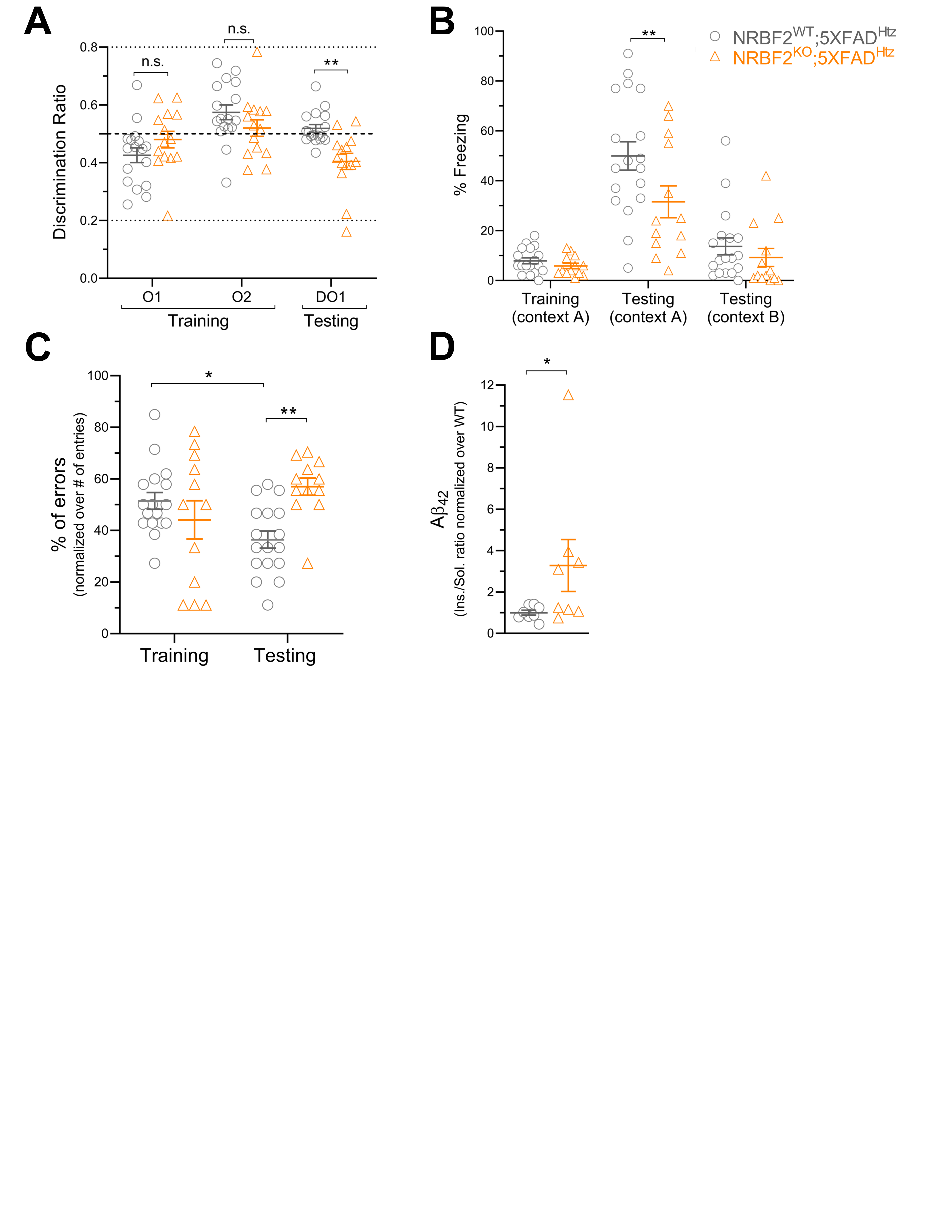


**Figure S5: Loss of NRBF2 in 5XFAD mice promotes memory deficits and enhances Aβ aggregation. (A)** Discrimination ratio obtained from OLT task. Results are mean±SEM of WT (n=17) and KO (n=13) mice. **(B)** Freezing behavior recorded during CFC experiments. Results are mean±SEM of WT (n=17) and KO (n=13) mice. **(C)** Percentage of errors measured during RAM experiments. Results are mean±SEM of WT (n=17) and KO (n=12) mice. The statistical significance was determined using row-matched two-way ANOVA test followed by Bonferroni’s post-test. *p<0.05, **p<0.01.  **(D)** Aβ_42_ insoluble over soluble ratio from ELISA analysis of human Aβ_42_ level in hippocampal Triton-X-100 soluble and insoluble fractions of NRBF2^WTorHtz^;5XFAD^Htz^ and NRBF2^KO^;5XFAD^Htz^ mice. Results are mean±SEM of WT (n=8) and KO (n=8) animals. The statistical significance was determined using one-tailed unpaired Student's t-test. *p<0.05.

**
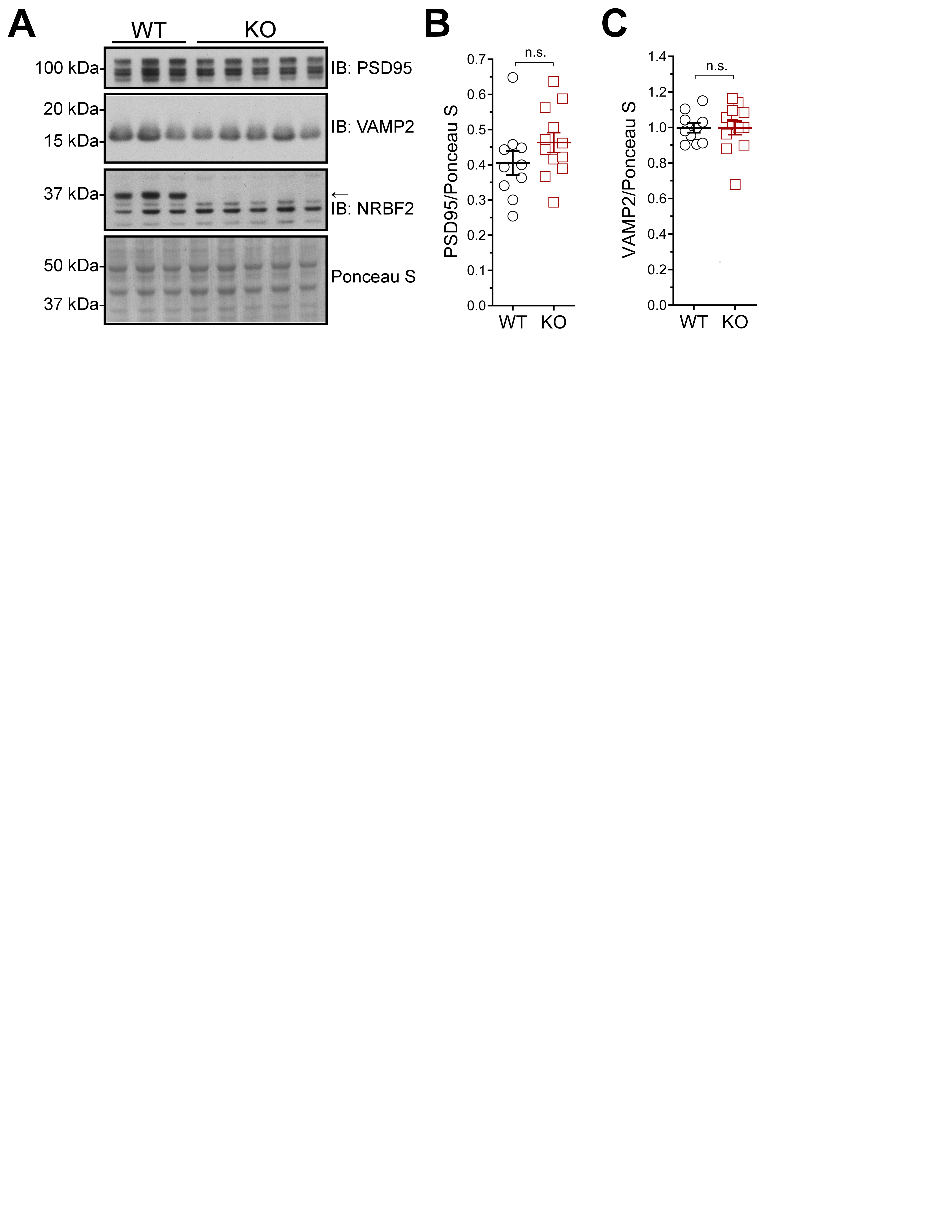
**

**Figure S6: Pre- and post-synaptic markers expression is unchanged in NRBF2-KO hippocampi. (A)** Immunoblot analysis of PSD95 and VAMP2 expression. The blots shown are representative of three separate experiments.  **(B-C)** Quantification of **A**. Results are mean±SEM of WT (n=10) and KO (n=12) mice. The statistical significance was determined using two-tailed unpaired Student's t-test.

**
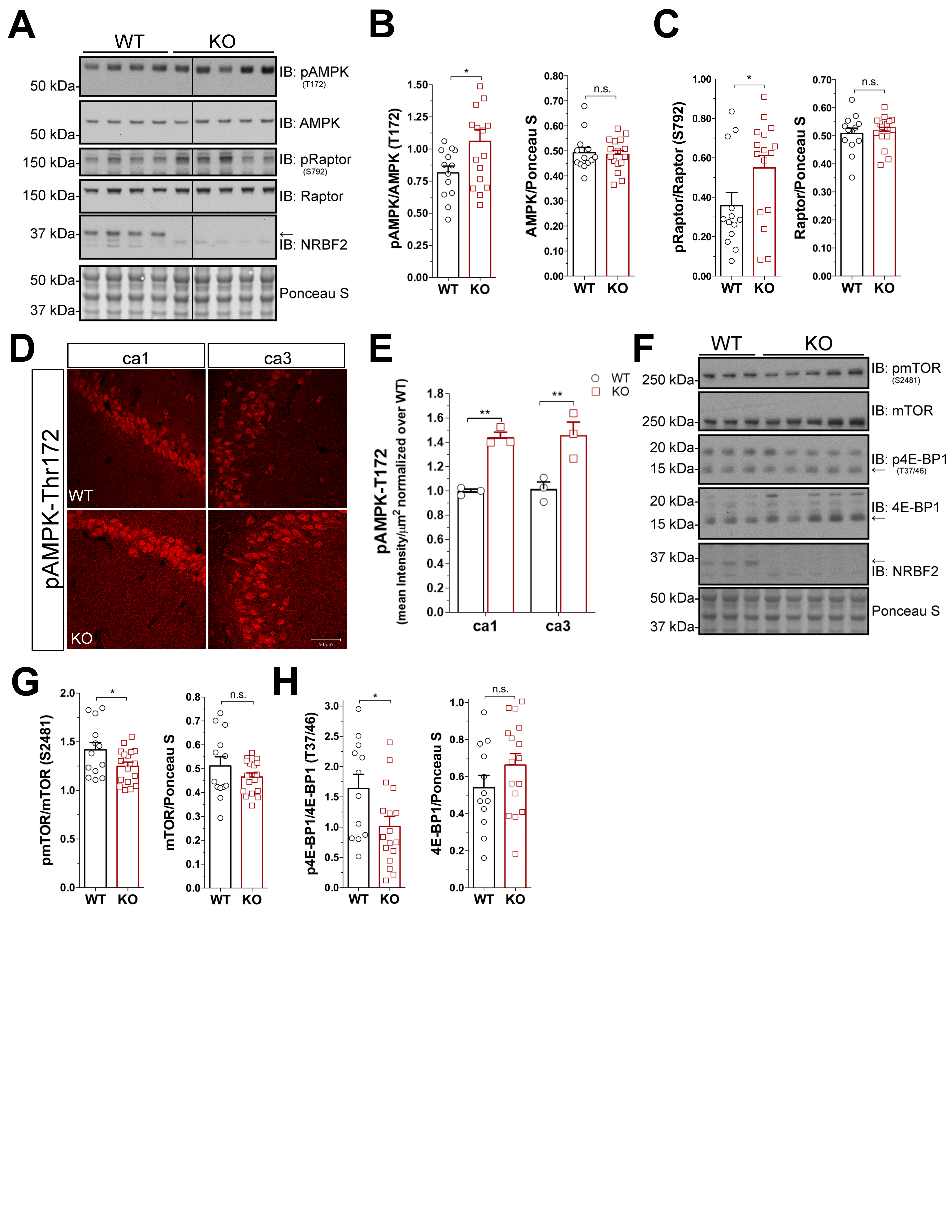
**

**Figure S7: Upregulation of AMPK activity inhibits mTORC1 signaling in hippocampus of NRBF2-KO mice. (A)** Immunoblot analysis of phospho-AMPK-Thr172, AMPK, phospho-Raptor-Ser792 and Raptor expression in hippocampal homogenates. The blots shown are representative of three separate experiments. **(B-C)** Quantification of **A**. Results are mean±SEM of WT (n=13) and KO (n=16) mice. The statistical significance was determined using two-tailed unpaired Student's t-test. **(D)** Immunofluorescence analysis of phospho-AMPK-Thr172 level in ca1 and ca3 hippocampal region of WT and NRBF2-KO mice. Scale bars, 50 μm. Images shown are representatives of two different experiments. **(E)** Quantification of **D**. Results are mean±SEM of WT and KO sections from three different mice. The statistical significance was determined using regular two-way ANOVA test followed by Bonferroni’s post-test. **(F)** Western blot analysis of phosho-mTOR-Ser2481, mTOR, phospho-4E-BP1-Ther37/46 and 4E-BP1 expressions in hippocampal homogenates. The blots shown are representative of three separate experiments. **(G-H)** Quantification of **F**. Results are mean±SEM of WT (n=12) and KO (n=16) mice. The statistical significance was determined using two-tailed unpaired Student's t-test. *p<0.05, ***p<0.001.


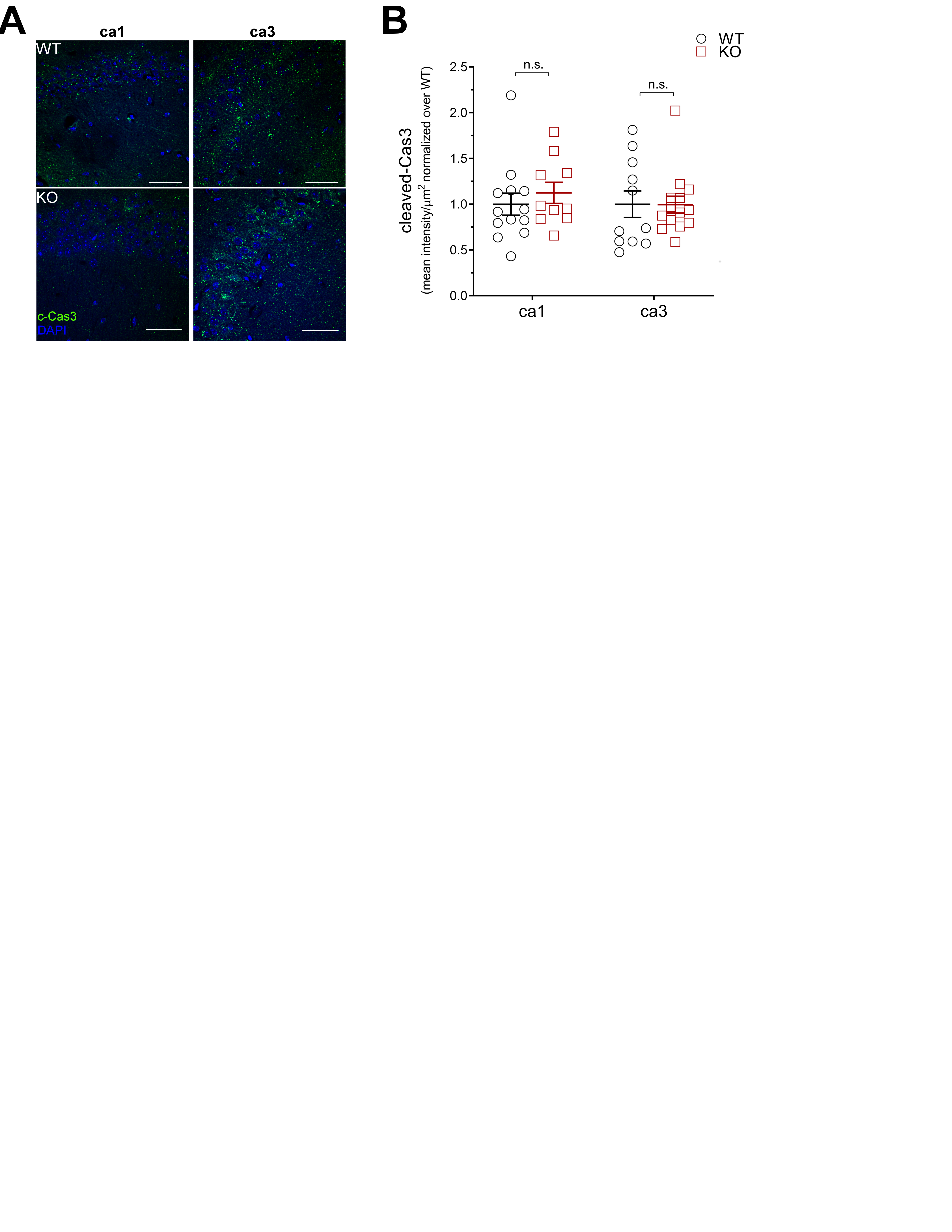


**Figure S8: Level of cleaved-caspase 3 is not changed in hippocampus of *NRBF2* KO mice. (A)** Immunofluorescence analysis of cleaved-Caspase3 level in ca1 and ca3 hippocampal region of WT and NRBF2-KO mice. Scale bars, 50 μm. Images shown are representatives of two different experiments. **(B)** Quantification of **A.** Results are mean±SEM of WT (ca1=14, ca3=12) and KO (ca1=11, ca3=14) sections acquired from four separate mice per group. The statistical significance was determined using regular two-way ANOVA test followed by Bonferroni’s post-test. *p<0.05, ***p<0.001.

**REFERENCES**

1. Lim J, Lachenmayer ML, Wu S, Liu W, Kundu M, Wang R, Komatsu M, Oh YJ, Zhao Y, Yue Z: **Proteotoxic stress induces phosphorylation of p62/SQSTM1 by ULK1 to regulate selective autophagic clearance of protein aggregates.** *PLoS genetics* 2015, **11:**e1004987.

2. Wold MS, Lim J, Lachance V, Deng Z, Yue Z: **ULK1-mediated phosphorylation of ATG14 promotes autophagy and is impaired in Huntington's disease models.** *Mol Neurodegener* 2016, **11:**76.

3. Schmittgen TD, Livak KJ: **Analyzing real-time PCR data by the comparative C(T) method.** *Nature protocols* 2008, **3:**1101-1108.

4. Shen EY, Jiang Y, Javidfar B, Kassim B, Loh YE, Ma Q, Mitchell AC, Pothula V, Stewart AF, Ernst P, et al: **Neuronal Deletion of Kmt2a/Mll1 Histone Methyltransferase in Ventral Striatum is Associated with Defective Spike-Timing-Dependent Striatal Synaptic Plasticity, Altered Response to Dopaminergic Drugs, and Increased Anxiety.** *Neuropsychopharmacology : official publication of the American College of Neuropsychopharmacology* 2016, **41:**3103-3113.
